# What Kills Mature Street and Park Trees in Cities? Systematic Quantitative Review of Published Case Studies

**DOI:** 10.1007/s00267-025-02116-2

**Published:** 2025-01-25

**Authors:** Anna Petrova, Ruby Naomi Michael, Chris Pratt

**Affiliations:** 1 Green Infrastructure Research Labs (GIRLS), Cities Research Institute, Griffith University, 170 Kessels Road, Nathan, 4111, QLD Australia; 2 School of Environment and Science, Griffith University, 170 Kessels Road, Nathan, 4111, Australia; 3 School of Engineering and Built Environment, Griffith University, Nathan, 4111, QLD Australia; 4 Australian Rivers Institute, Griffith University, 170 Kessels Road, Nathan, 4111, QLD Australia

**Keywords:** City, Decline, Dieback, Tree, Urban

## Abstract

Street and park trees often endure harsher conditions, including increased temperatures and drier soil and air, than those found in urban or natural forests. These conditions can lead to shorter lifespans and a greater vulnerability to dieback. This literature review aimed to identify confirmed causes of street and park tree dieback in urban areas from around the world. Peer-reviewed case studies related to urban tree decline were scanned for the words “urban”, “city”, “cities”, “tree*”, “decline”, “dieback”, “mortality”, and “survival”. From an initial pool of 1281 papers on Web of Science and 1489 on Scopus, 65 original peer-reviewed research papers were selected for detailed analysis. Out of all species reported to decline, 46 were native, while non-natives were represented by 35 species. The most commonly affected trees were *Platanus, Fraxinus, Acer, and Ficus*. Most studies were conducted in Mediterranean, humid subtropical, and humid continental climates, with the greatest representation from the United States, followed by Australia, Brazil, Iran, Italy, and Russia. Many authors focused on either biotic or abiotic causes of dieback; some explored both, and some also discussed underlying environmental and urban stresses as potential predisposing factors. The majority (81% of the papers) concluded that a decline was caused by either an arthropod or a microorganism. Overall, it was suggested that changing management strategies to improve water availability and soil health might help with tree resilience. Additionally, regular monitoring and research, along with improving tree species selection and implementing biological and chemical control methods, can help prevent or slow down tree decline. Increasing awareness and adopting preventative approaches could help to extend the lifespan of street and park trees in urban environments and mitigate some of the biological threats, especially considering the challenges we may be facing due to the changing climate.

## Introduction

### Urban Environments

Urban development dramatically alters ecosystems. Cities with large areas of hard surfaces are hotter than natural ecosystems and experience the Urban Heat Island effect (UHI) (Yang et al. 2016). This changes material and energy flow and affects air and soil temperature, hydrology, biological habits, and human health. Not only are cities hot, but they are also dry. Due to the loss of vegetation, atmospheric humidity in urban areas is lower while the vapour pressure deficit is higher, which creates the Urban Dry Island effect (UDI) (Lu et al. 2018). This results in a drop in soil moisture, as well as in average intensity of UDI of −7.2% over one year, according to Shi et al. (Shi et al. 2012).

The need for roads and paths in cities results in a high cover of impermeable surfaces (Lal and Stewart 2017); evapotranspiration here decreases, while runoff increases (Wessolek 2008). In addition to contributing to the UHI effect, pavements create an inhospitable environment for tree roots, resulting in a shorter tree life span (Smiley et al. 2006) by reducing the infiltration of precipitation (Randrup et al. 2001).

As cities expand and become more densely populated, much of the natural vegetation is lost (Richards and Belcher 2019), and new plant species are often introduced. When selecting trees for these urban areas, hardy varieties are typically chosen, with non-native species frequently planted. However, there is an ongoing debate about whether to continue planting non-native trees. Some advocate for a “native-only” approach, arguing that native species offer greater ecosystem services (Arcos-LeBert et al. 2021), support larger insect populations (Helden et al. 2012), and contribute to ecosystem integrity and adaptability (de Carvalho et al. 2022). On the other hand, some believe that introduced species are essential and cannot be excluded (Sjöman et al. 2016).

### Tree Failure

Urban tree dieback is a scenario where a rapid decline is observed in trees exhibiting similar symptoms and located nearby. Symptoms may include foliage discolouration or loss, cankers, and branch or whole tree dieback. When this happens, the event must be investigated, and a treatment must be found to protect other, still unaffected trees.

Tree resilience is significantly shaped by both ecological conditions and urban activities. Stressors such as traffic, inadequate maintenance, and physical damage can also contribute to tree dieback and general decline (Tello et al. 2005). Yet, the critical determinant of a tree’s ability to withstand such stresses lies in its environment. Decades ago, Patterson (1977) noted that 90% of urban tree problems relate to soil health, while Sanders and Grabosky (2014) later confirmed that stunted tree growth is consistently associated with lower soil quality, which can include compaction, nutrient loads, and percentage of organic matter (Day and Bassuk 1994; Scharenbroch et al. 2005).

Urban trees are not a cheap asset. Direct and indirect costs are associated with their management - from purchasing a tree and labour costs during planting, mulching, and initial maintenance to pruning, diagnostics, and pest and disease control. Research conducted by Moore (2022) shows that, for example, in Australia, maintenance for a single tree over 50 years will cost councils between $2800 and $6220. Scaled up across entire urban forests in cities, this is a substantial cost.

This systematic quantitative literature review aimed to understand what affects tree vitality in cities and to outline urban tree management strategies. Therefore, the review looked into published case studies of urban tree decline from around the world to answer the following questions: (a) What tree species are commonly affected? (b) Does endemicity affect the frequency of decline? (c) What are the most common causes of tree dieback (d) What management strategies can be applied to prevent failure?

## Methods

### Search Methodology

The review was conducted based on the method described by Pickering and Byrne (2014). Peer-reviewed articles written in English were extracted from *Thompson ISI Web of Science and Scopus* in July 2024. For both databases, the search terms used to capture studies related to urban tree health in title, abstract, and keywords were: “urban”, “city”, “cities”, “tree*”, “decline”, “dieback”, “mortality”, and “survival” and were limited to articles only. The word “peri-urban” was excluded from the search. In the Web of Science, the document types were limited to the following categories: Environmental Sciences, Forestry or Ecology, Environmental Studies, Plant Sciences, Urban Studies, and Biodiversity Conservation. In Scopus, the selected categories were Environmental Science and Agricultural and Biological Sciences. Reports from arborists for various councils were read but are not incorporated as this review focuses exclusively on peer-reviewed literature.

Publication abstracts were screened for these terms and shortlisted. Only papers written in English discussing mature and established trees in small urban parks and street trees were selected, as they better reflect the impacts of urbanisation. Reference lists in those papers were screened to find publications that were not captured by the databases. Studies involving greenhouse experiments, research on seedlings, or investigations conducted in extensive urban forests were excluded.

### Data Classification

The short-listed papers were read in their entirety, with specific information extracted and entered into an Excel database. The following information was captured and included: (I) citation information, (II) year of the study, (III) country of the study, (IV) climate, (V) tree species, (VI) tree origin, (VII) year dieback was first observed, (VIII) symptoms in trees, (IX) main cause of dieback, (X) secondary or underlying cause of dieback, (XI) pests/pathogens if present, and (XII) proposed management.

### Data Visualisation

The locations were visualised on a world map to show the spatial distribution of the studies identified for this review. Bar and scatter plots were created in Excel to demonstrate the distribution of the quantity of the research conducted in those countries, the climates reflected in the studies, and the years of publication. Information related to tree species affected, their causes of decline, biotic and abiotic, was presented in tables while contributing factors were shown as a word cloud created in RStudio using the wordcloud2 library.

## Results

**Fig. 1:**
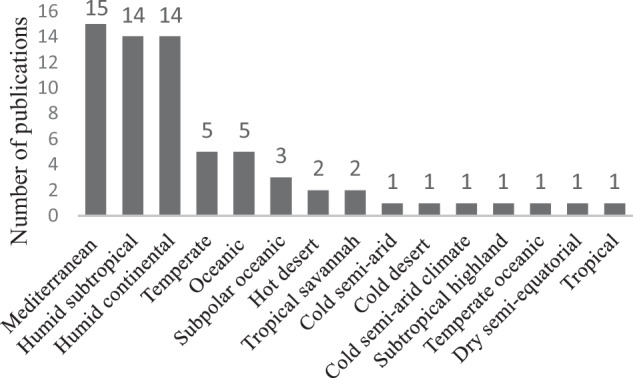
Climates presented in the studies, arranged in order from highest to lowest

In total, 1281 articles from the Web of Science and 1687 from Scopus were assessed for inclusion, of which 65 articles were selected and read as a whole for this study. The majority of the studies analysed were conducted in countries with Mediterranean, humid subtropical, and humid continental climates (Fig. 1).

**Fig. 2:**
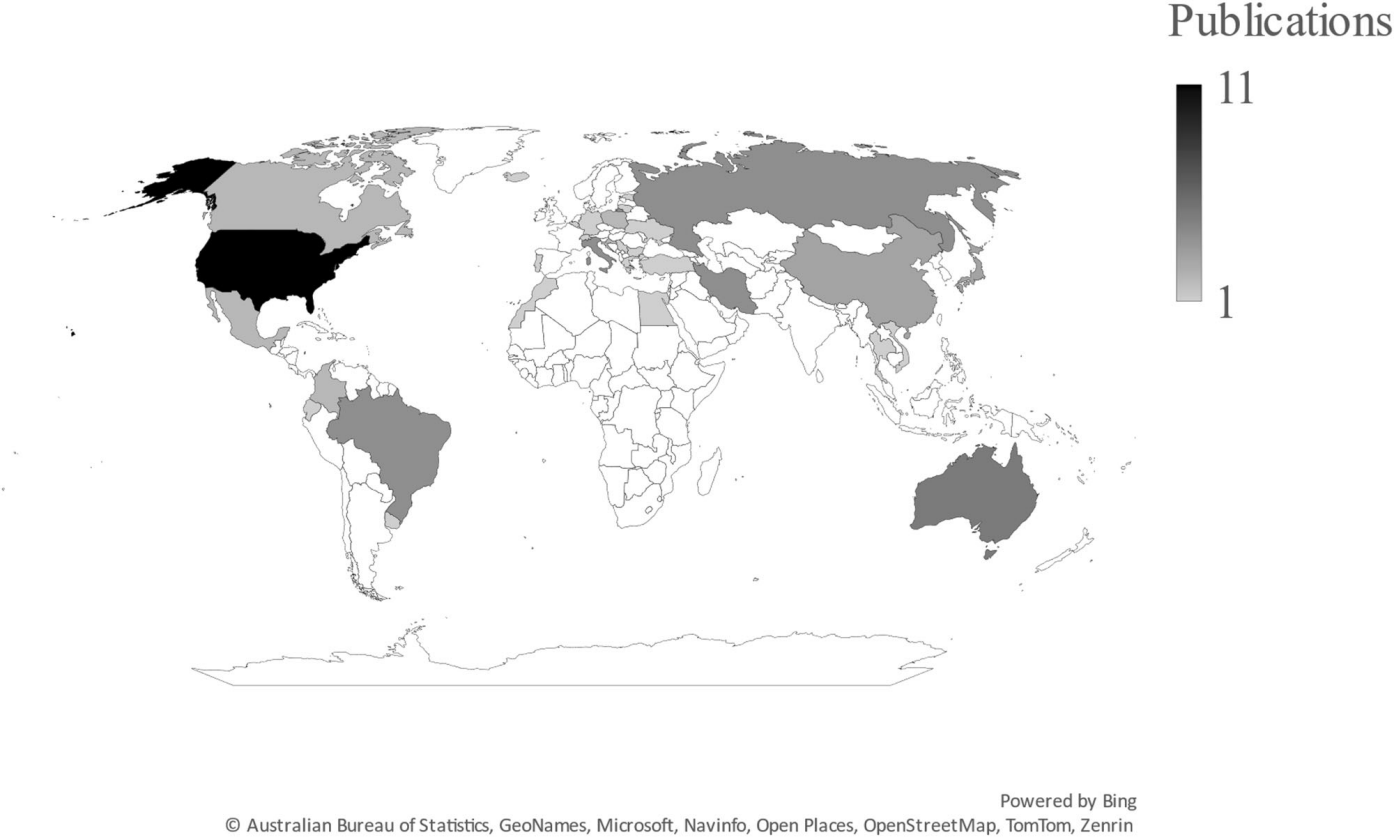
Countries represented in the studies. The darker colours show countries with the higher output numbers

The highest number of studies were conducted in the USA, followed by Australia, Brazil, Iran, Italy, Russia, and other countries (Fig. 2, Table 1).

**Table 1:** Main causes of tree decline, affected tree species, and factors predisposing or contributing to tree decline

Main causes of tree decline	Number of publications	Publications	Tree species	Predisposing/contributing factors
Strong wind	3	(a) Das Graças Emerick et al., (2024); (b) Fukui et al. (2018); (c) Jim (2005)	(a) *Spathodea campanulata P. Beauv.;* (b) *Ulmus davidiana var. japonica, Populus sieboldii, Ailanthus altissima (Mill.) Swingle, Robinia pseudoacacia Linn.;* (c) multiple, heritage	(a) Interference with a powerline, sparse foliage, overgrowth onto pavement or sidewalk, presence of fungi in a trunk and a base, form not neiloid; (b) Presence of wood-decay fungi (*Polyporus squamous*); (c) Injuries from roadworks and road construction resulting in the disturbance of soil and root damage
Water stress	2	(a) May et al. (2013); (b) Takamatsu et al. (2001)	(a) *Platanus × acerifolia, Ulmus procera ;* (b) *Cryptomeria japonica*	(a) Drought; (b) Stomata clogging with aerosols (air pollution) and environmental aridification
Heat stress	2	(a) Betzen et al. (2021); (b) Marchin et al. (2022)	(a) *Acer macrophyllum;* (b) multiple species	(a) Proximity to roads and urban environment; (b) Plant functional traits
Saltwater flooding	1	Hallett et al. (2018)	*Platanus×acerifolia, Acer rubrum*	-
Mechanical stability failure	1	Korniyenko and Kalaev (2022)	*Betula pendula* Roth	Anthropogenic load, high wind, low temperatures, freezing rain, thawing
Flood and incorrectly selected species	1	Leksungnoen (2017)	Multiple species	Plant functional traits
Natural gas leaks (methane)	1	Schollaert et al. (2020)	Multiple species	-
Manganese deficiency	1	Grigg et al. (2009)	*Eucalyptus marginata, Corymbia calophylla*	Reduced plant availability of Mn due to irrigation with alkaline water which increased soil pH

**Fig. 3:**
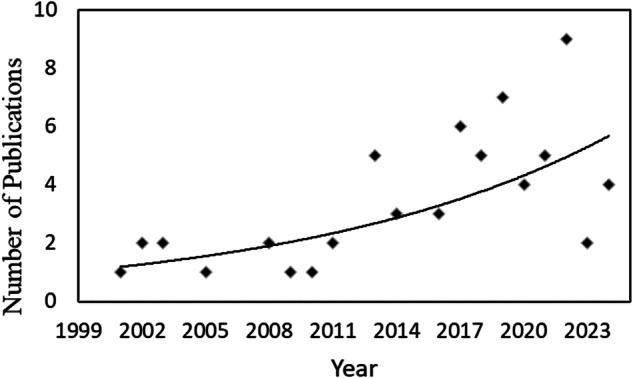
Increase in the number of publications on urban street and park tree dieback in the last 25 years, modelled by exponential regression (*P* < 0.05)

The earliest study found and related to urban street and park tree dieback was published in 2001, and until 2013, the publication output was limited to between 0 and 1 paper per year. Interest in the topic started to grow in 2013, although it fluctuated from year to year. The highest number of research output in a year was nine publications in 2022 and 6 and 7 publications in 2017 and 2019, respectively (Fig. 3).

### Affected Tree Species

Species selection was largely dependent on the country where each study was conducted. The most commonly investigated genera were *Platanus, Fraxinus, Acer, and Ficus*. Other genera included *Pinus, Populus*, *Ulmus*, *Aesculus*, *Abies*, *Celtis*, *Picea*, *Salix*, and *Tilia*. Thirteen other species were represented by one study each (Table 2). Eight additional studies investigated the dieback of multiple tree species, and two described trees as ‘heritage’. More than half of the trees were native (46), compared with 35 exotic species.

**Table 2:** Families and species of the fungal pathogens discussed in the publications, tree species affected, and predisposing/contributing factors discussed by the authors

Family	Species	Number of publications	Affected trees	Predisposing/contributing factors	Publications
Botryosphaeriaceae	*Botryosphaeria dothidea*	3	*Sequoia sempervirens, Ficus macrocarpa, Sequoiadendron giganteum* Lindl.	Water stress, droughts (Acimovic et al. 2018; Kovac et al. 2021), climate change, warming climate, disturbed interactions between wood-inhabiting fungi (Fiorenza et al. 2022)	Acimovic et al. (2018); Fiorenza et al. (2022); Kovac et al. (2021)
	*Neofusicoccum australe*	1	*Sequoia sempervirens*	Water stress, droughts (Acimovic et al. 2018)	Acimovic et al. (2018)
	*Neofusicoccum luteum*	1	*Sequoia sempervirens*	Water stress, droughts (Acimovic et al. 2018)	Acimovic et al. (2018)
	*Neofusicoccum mediterraneum*	2	*Sequoia sempervirens, Ficus microcarpa*	Water stress, droughts (Acimovic et al. 2018)	Acimovic et al. (2018); Fiorenza et al. (2022)
	*Neofusicoccum parvum*	4	*Sequoia sempervirens, Ficus macrocarpa, Platanus × acerifolia*	Water stress, droughts (Acimovic et al. 2018; Pelleteret et al. 2017), climate change, warming climate, disturbed interactions between wood-inhabiting fungi (Fiorenza et al. 2022)	Acimovic et al. (2018); Fiorenza et al. (2022); Pelleteret et al. (2017); Scattolini Rimada et al. (2023)
	*Neofusicoccum yunnanense*	1	*Sequoiadendron giganteum* Lindl.	Water stress, droughts (Kovac et al. 2021)	Kovac et al. (2021)
	*Diplodia mutila*	2	*Platanus × acerifolia*	Water stress (Pelleteret et al. 2017)	Pelleteret et al. (2017); Scattolini Rimada et al. (2023)
	*Dothiorella sp*.	1	*Platanus × acerifolia*	Water stress (Pelleteret et al. 2017)	Pelleteret et al. (2017)
	*Diplodia seratia*	1	*Platanus × acerifolia*	Water stress (Pelleteret et al. 2017)	Pelleteret et al. (2017)
	*Diplodia mutila*	1	*Platanus × acerifolia*	Water stress (Pelleteret et al. 2017)	Pelleteret et al. (2017)
	*Diplodia pseudoseriata*	1	*Platanus × acerifolia*	-	Scattolini Rimada et al. (2023)
	*Neoscytalidium novaehollandiae*	1	*Pinus eldarica*	Higher temperature, low humidity (Alizadeh et al. 2022),	Alizadeh et al. (2022)
	*Lasiodiplodia theobromae*	2	*Ficus benghalensis L, Ficus nitida, Ficus hawaii*	Environmental factors, unsuitable climate, poor soil, large portion of rootzone under pavement, inadequate irrigation, excessive pruning, root pruning (Abo Rehab et al. 2014), water deficiency, drought conditions, arthropods as a vector, tree management, pruned debris on the ground act as inoculum (Yeganeh and Mohammadi 2022)	Abo Rehab et al. (2014); Yeganeh and Mohammadi (2022)
	*Neoscytalidium dimidiatum*	1	*Ficus benghalensis L*	Water deficiency, drought conditions, arthropods as a vector, tree management, pruned debris on the ground act as inoculum (Yeganeh and Mohammadi 2022)	Yeganeh and Mohammadi (2022)
	*Sardiniella urbana gen. et sp. nov*.	1	*Celtis australis*	-	Linaldeddu et al. (2016)
	*Phyllosticta paviae Desm. (Guignardia aesculi (Peck) V.B. Stewart)*	1	*Aesculus hippocastanum, Aesculus × carnea*	Water stress, urbanisation, traffic pollution, *Cameraria ohridella* (Dziegielewska et al. 2017)	Dziegielewska et al. (2017)
Hymenochaetaceae	*Inonotus rickii*	1	*Acer negundo, Celtis australis*	Higher temperatures (Annesi et al. 2003)	Annesi et al. (2003)
Rhytismataceae	*Rhytisma acerinum*	1	*Acer platanoides*	-	Lapointe and Brisson (2011)
Dothioraceae	*Sydowia polyspora*	1	*Abies concolor*	Climate change, high summer temperatures, droughts (Lazarevic and Menkis 2022)	Lazarevic and Menkis (2022)
	*Aureobasidium pullulans*	1	*Tilia cordata Mill*., multiple species	High salt concentrations in soil (Snieskiene et al. 2016)	Snieskiene et al. (2016)
Ophiostomataceae	*Ophiostoma ulmi*	1	*Ulmus davidiana var. japonica*	Higher temperature, low precipitation, soil compaction (Miyamoto et al. 2019)	Miyamoto et al. (2019)
	*Ophiostoma novo-ulmi*	1	*Ulmus davidiana var. japonica*	Higher temperature, low precipitation, soil compaction (Miyamoto et al. 2019)	Miyamoto et al. (2019)
Nectriaceae	*Fusarium decemcellulare Brick*	1	*Mangifera indica*	-	Qi et al. (2013)
	*Fusarium circinatum*	2	*Pinus radiata*	Arthropods as vector (Storer et al. 2002), landscape type, location, weather (Wikler et al. 2003)	Storer et al. (2002); Wikler et al. (2003)
	*Fusarium oxysporum*	1	*Fortunella margarita cv. Guba*	Human activities intensify movement of inoculum, altered biotic interactions, reduced evaporative demand, presence of an arthropod as a vector (Zhu et al. 2013)	Zhu et al. (2013)
	*Fusarium sp. GLB1*	1	*Fortunella margarita cv. Guba*	Human activities intensify movement of inoculum, altered biotic interactions, reduced evaporative demand, presence of an arthropod as a vector (Zhu et al. 2013)	Zhu et al. (2013)
	*Fusarium solani*	1	*Platanus × acerifolia*	Drought, tree spacing, suppression by other species, poor soil drainage, pruning, genetic susceptibility (Pilotti et al. 2002)	Pilotti et al. (2002)
Aspergillaceae	*Aspergillus brasiliensis*	1	*Tilia cordata Mill*., multiple species	High salt concentrations in soil (Snieskiene et al. 2016)	Snieskiene et al. (2016)
Davidiellaceae	*Cladosporium herbarum*	1	*Tilia cordata Mill*., multiple species	High salt concentrations in soil (Snieskiene et al. 2016)	Snieskiene et al. (2016)
Helotiaceae	*Hymenoscyphus fraxineus*	2	*Fraxinus excelsior*	-	Shabunin (2020); Volke et al. (2019)
Valsaceae	*Cytospora friesii*	1	*Abies concolor*	Climate change, high summer temperatures, droughts (Lazarevic and Menkis 2022)	Lazarevic and Menkis (2022)
	*Phomopsis spp*.	2	*Platanus orientalis, Platanus × acerifolia, Ficus benghalensis L, Ficus nitida, Ficus hawaii*	Environmental factors, unsuitable climate, poor soil, large portion of rootzone under pavement, inadequate irrigation, excessive pruning, root pruning (Abo Rehab et al. 2014)	Georgieva et al. (2023); Abo Rehab et al. (2014)
	*Cytospora platani*	1	*Platanus orientalis, Platanus × acerifolia*	-	Georgieva et al. (2023)
Erysiphaceae	*Erysiphe flexuosa*	1	*Aesculus hippocastanum, Aesculus × carnea*	Water stress, urbanisation, traffic pollution, *Cameraria ohridella* (Dziegielewska et al. 2017)	Dziegielewska et al. (2017)
Polyporaceae	*Polyporus squamosus*	1	*Populus squamosus*	Extreme weather, pruning wounds (Fukui et al. 2018)	Fukui et al. (2018)
Gnomoniaceae	*Apiognomonia veneta*	1	*Platanus orientalis, Platanus × acerifolia*	-	Georgieva et al. (2023)
Physalacriaceae	*Armillaria mellea*	1	*Platanus orientalis, Platanus × acerifolia*	-	Georgieva et al. (2023)
Erysiphaceae	*Erysiphe platani*	1	*Platanus orientalis, Platanus × acerifolia*	-	Georgieva et al. (2023)
Ceratocystidaceae	*Ceratocystis platani*	1	*Platanus orientalis, Platanus × acerifolia*	Tool contamination, contaminated wood dust, pruning of the diseased trees, dead trees left in-situ, tree spacing (Lehtijärvi et al. 2018)	Lehtijärvi et al. (2018)
Ophiostomataceae	*Ophiostoma novo-ulmi subsp. novo-ulmi*	1	*Ulmus spp*.	-	Jürisoo et al. (2019)
	*Ophiostoma novo-ulmi subsp. Americana*	1	*Ulmus spp*.	-	Jürisoo et al. (2019)
Sporocadaceae	*Pestalotiopsis pini sp. nov*.	1	*Pinus pinea L*.	Water stress, air temperature (Silva et al. 2020)	Silva et al. (2020)
	*Pestalotiopsis biciliata*	1	*Platanus × acerifolia*	-	Scattolini Rimada et al. (2023)
	*Pestalotiopsis rhodomyrtus*	1	*Platanus × acerifolia*	-	Scattolini Rimada et al. (2023)
Glomerellaceae	*Colletotrichum acutatum*	1	*Platanus × acerifolia*	-	Scattolini Rimada et al. (2023)

### Causes of Tree Dieback

Most studies only investigated one main potential cause of decline, either biotic or abiotic, although many have discussed possible secondary causes or predisposing factors. Pathogens or pests were identified as a primary cause of dieback in the majority of studies (81%, *n* = 52) (Table 3). Only 19% of the studies found an abiotic factor to be the main reason for tree decline. In most of those cases, neither pathogens nor pests were investigated.

**Table 3:** Families and species of oomycetes discussed in the publications, trees they affected, and predisposing/contributing factors

Family	Species	Number of publications	Affected trees	Predisposing/contributing factors	Publications
Phytophthora	*Phytophthora cinnamomi*	1	*Platanus × acerifolia, Platanus orientalis*	Climate change, infection from nursery plants (Antonelli et al. 2024)	Antonelli et al. (2024)
	*Phytophthora nicotianae*	1	*Platanus × acerifolia, Platanus orientalis*	Climate change, infection from nursery plants (Antonelli et al. 2024)	Antonelli et al. (2024)
	*Phytophthora mediterranea*	1	*Platanus × acerifolia, Platanus orientalis*	Climate change, optimal temperature for growth, infection from nursery plants (Antonelli et al. 2024)	Antonelli et al. (2024)
	Multiple species	1	Multiple species	Globalisation (Khdiar et al. 2020)	Khdiar et al. (2020)

#### Abiotic Factors

Two studies looked at the effect of water availability on tree health (Table 3). In Japan, Takamatsu et al. (2001) concluded that chronic water stress in *Cryptomeria japonica* and its decline was likely caused by the deterioration of epicuticular wax and stomata clogging with aerosols from the gaseous air pollutants, in addition to the environmental aridification. In Melbourne, Australia, May et al. (2013) investigated the effects of drought between 1997 and 2009 on multiple tree species. They found that extended water stress was a substantial problem but noted that the nature of tree decline is multifactorial, and additional stresses like elm leaf beetle infestation had an impact. They also noted that the choice of the season for drip irrigation was important for its effectiveness, and late winter was the best time for increasing soil moisture levels. Trees that showed a lesser level of decline originated from drier regions of Australia.

Mechanical damage due to strong winds was a topic of three publications. Jim (2005) discussed Hong Kong’s harsh tree habitat conditions, which are caused by rapid development and high population density. While the loss of 54 out of 380 heritage trees there between 1993–1998 and 1999–2003 was related to typhoon tree breakage, the trees were predisposed to the decline due to injuries they sustained during various construction and road works, which resulted in soil disturbance and root damage. Das Graças Emerick et al. (2024) also found that the risk of damage was higher when there was a tree interference with a powerline, sparse foliage, overgrowth onto pavement or sidewalk, presence of fungi in a trunk and a base, and a form of a tree. Finally, Fukui et al. (2018) noticed the presence of wood-decay fungi in the damaged trees.

The problem of heat stress was addressed in two studies. In the US, Betzen et al. (2021) found that *Acer macrophyllum* decline was positively associated with increased development, proximity of roads, and rising summer temperatures. No biotic agents affecting tree health were identified. In Australia, Marchin et al. (2022) concluded that heat rather than drought stress was a threat to urban trees during the hot summer of 2019–2020 and that plant functional traits had a role in the decline.

New York City lost over 20,000 street trees due to Hurricane Sandy, which landed in 2012 (Hallett et al. 2018). Saltwater flooding followed this event and was the cause of the decline of 48,000 more trees in the urban coastal forest. It was found that crowns of a significant proportion of species like London plane *(Platanus×acerifolia*) and maple (*Acer* spp.) failed to leaf out. The trees were found to be not suitable for the flooded areas and experienced high mortality.

Significant temperature changes, creating freezing and thawing events, may affect the biomechanical parameters of a tree. Korniyenko and Kalaev (2022) investigated the failure of the silver birch tree (*Betula pendula* Roth) in Donetsk, Ukraine and studied the impact of temperature on its woody-tissue elastic modulus. They found that the elasticity modulus decreases, on average, 2–2.5 times at thawing temperatures, and together with the anthropogenic pressure and wind loads, as well as snow and ice storm conditions, causing irreversible damage to the trees.

The 2011 flood in Bangkok, Thailand, resulted in prolonged inundation which, in some areas, lasted a month (Leksungnoen 2017). After the event, species richness in the area was decreased by 18%. While most of the highly flood-tolerant species and some species native to drier, often deciduous lowland habitats recovered after the event, losses were high for Magnoliaceae and Lauraceae families.

A study conducted by Schollaert et al. (2020) in Massachusetts, USA, looked at the natural gas levels in tree pits of declining and healthy trees. It was found that those in poor health were exposed to significantly higher methane levels in the soil and therefore were possibly growing in anaerobic soil conditions. The concentrations of the gas were highest on the side of trees that were closest to the road, indicating that there was possible methane leakage from the underground urban gas distribution systems.

While most studies looked at climatic or environmental conditions, a few found less common reasons for tree decline, such as nutrient deficiencies, construction works trauma, soil gas levels and extreme weather events. In Australia, Grigg et al. (2009) found manganese deficiencies in parkland tree foliage. Given that those trees were irrigated with alkaline water, they concluded that alkalinity reduced the availability of manganese. The authors point out that they only looked at the trees in one park and were therefore unable to draw wide conclusions.

#### Biotic Factors (pathogens/pests)

##### Fungal Pathogens

Among pathogenic fungi, 17 species from the Botryosphaeriaceae family were by far the leading cause of decline and were found in twelve tree families (Table 4). The symptoms of these pathogens included yellowing and wilting of leaves, defoliation, chlorosis, necrosis, needle dieback, cankered twigs and branches, cork canker, internal wood discolouration, stem exudates, and bark damage (Abo Rehab et al. 2014; Alizadeh et al. 2022; Fiorenza et al. 2022; Linaldeddu et al. 2016; Pelleteret et al. 2017; Yeganeh and Mohammadi 2022). Nearly all studies associated the presence of Botryosphaeriaceae species with water stress, and many noted higher than usual temperatures.

**Table 4:** Families and species of bacteria discussed in the publications, trees they affected, and predisposing/contributing factors discussed by the authors

Family	Species	Number of publications	Affected trees	Predisposing/contributing factors	Publications
Xanthomonadaceae	*Xylella fastidiosa*	2	*Salix alba L., Populus nigra L*., multiple species	Water stress, xylem-feeding insect vectors (Harris et al. 2014)	Harris et al. (2014); Hashemi and Mohammadi (2016)
Acholeplasmataceae	*Candidatus Phytoplasma fraxini*	2	*Fraxinus uhdei, Liquidambar styraciflua*	Infected parental trees (Franco-Lara et al. 2017)	Filgueira et al. (2018); Franco-Lara et al. (2017)
	*Candidatus Phytoplasma asteris*	2	*Liquidambar styraciflua, Populus nigra L*.	Infected parental trees (Franco-Lara et al. 2017), irrigation levels, insect vectors (Music et al. 2008)	Franco-Lara et al. (2017); Music et al. (2008)
	*Candidatus Phytoplasma solani*	1	*Liquidambar styraciflua*	Infected parental trees (Franco-Lara et al. 2017)	Franco-Lara et al. (2017)
	*Phytoplasmas from 16SrIV-D subgroup*	1	*Phoenix canariensis*	Insect vectors (Ortiz-García et al., 2024)	Ortiz-García et al. (2024)
Rhizobiaceae	*Liberibacters genus*	1	*Murraya exotica*	Lack of regulation in production and commercialisation (Lopes et al. 2010)	Lopes et al. (2010)
Yersiniaceae	*Gibsiella quercinecans*	1	*Tilia cordata Mill*.	Insect vectors (Tkaczyk et al. 2024)	Tkaczyk et al. (2024)

Pathogens from the Nectriaceae family were represented by five species and were found in four tree families. The presence of these fungi caused wilting, dry leaves and defoliation, chlorotic needles, large irregular brown-coloured speckles on the petioles and twigs, dead branches, vascular necrosis, and canker (Qi et al. 2013; Storer et al. 2002; Zhu et al. 2013). Some of the contributing factors were human activity, altered biotic interactions, and arthropods acting as vectors, as well as drought, tree spacing, suppression by other species, poor soil drainage, pruning, and genetic susceptibility (Table 4).

All other fungal pathogens were less common with between one and three species identified. Valsaceae pathogens affected three tree families and were expressed as stem canker (Abo Rehab et al. 2014; Lazarevic and Menkis 2022). Sporocadaceae were found in two tree families and caused shoot blight and stem necrosis (Scattolini Rimada et al., 2023; Silva et al., 2020). The presence of species from Dothioraceae family led to foliage/needle decolouration, defoliation, and general dieback (Lazarević and Menkis 2022; Snieškienė et al. 2016) while infection with Ophiostomataceae species resulted in wilting, xylem browning, and were associated with the presence of *Scolytus esuriens* (elm bark beetle) (Miyamoto et al. 2019). Species from Aspergillaceae, Ceratocystidaceae, Cytosporaceae, Davidiellaceae, Diaporthaceae, Erysiphaceae, Glomerellaceae, Gnomoniaceae, Helotiaceae, Hymenochaetaceae, Polyporaceae, Physalacriaceae, and Rhytismataceae families were each identified once, and, similar to the other pathogens, caused foliage/needle discolouration, defoliation, wilting, chlorosis, necrosis, sooty layer on bark, and general dieback (Abo Rehab et al. 2014; Snieškienė et al. 2016; Volke et al. 2019).

##### Oomycetes

There was a limited number of studies related to oomycetes. Only two identified Phytophthora as a pathogen causing tree dieback (Table 5), yet in the natural environment, Phytophthora species frequently receive attention due to its severity. Antonelli et al. (2024) isolated *Phytophthora cinnamomi*, *Phytophthora nicotianae*, and *Phytophthora mediterranea* from Platanus species while Khdiar et al. (2020) investigated the impact of different land management strategies in urban parks and associated Phytophthora communities. From 234 samples collected, forty-four Phytophthora phylotypes were detected, with *P. multivora, P. arenaria, P. amnicolaand, and P. cinnamomi* found more frequently than other species. Climate change, optimal temperatures for growth, planting infected nursery trees and globalisation were identified as potential predisposing factors for the pathogen introduction and attack.

**Table 5:** Families and species of pests discussed in the publications, trees they affected, and predisposing/contributing factors discussed by the authors

Family	Species	Number of publications	Affected trees	Predisposing/contributing factors	Publications
Buprestidae	*Agrilus planipennis*	4	*Fraxinus* *pennsylvanica Marsh., Fraxinus excelsior, Fraxinus spp*.	Waterlogging, drought, frost, pests and diseases (Straw et al. 2013), higher temperatures (Volkovitsh et al. 2021)	Persad and Tobin (2015); Selikhovkin et al. (2022); Straw et al. (2013); Volkovitsh et al. (2021)
Diaspididae	*Melanaspis tenebricosa*	2	*Acer rubrum*	Higher temperatures, water stress (Backe et al. 2019), higher temperatures, water stress, water potential, urban heat island effect, soil compaction (Dale and Frank 2014)	Backe et al. (2019); Dale and Frank (2014)
Coccoidea	*Crypticerya multicicatrices* Kondo & Unruh	1	Multiple species	Trade of infected plants, ineffective pest control in nurseries (de López et al. 2022)	de López et al. (2022)
	*Crypticerya genistae* (Hempel)	1	Multiple species	Trade of infected plants, ineffective pest control in nurseries (de López et al. 2022)	de López et al. (2022)
Curculionidae	*Euwallacea whitfordiodendrus*	1	Multiple species	Natural flight dispersal, movement of green waste from urban areas, movement of firewood and nursery stock, diverse host range (Coleman et al. 2019)	Coleman et al. (2019)
	*Euwallacea kuroshio Gomez and Hulcr*	1	Multiple species	Natural flight dispersal, movement of green waste from urban areas, movement of firewood and nursery stock, diverse host range (Coleman et al. 2019)	Coleman et al. (2019)
Aphididae	*Elatobium abietinum*	1	*Picea sitchensis*	Higher winter temperatures, elevated N in tree needles due to car traffic, proximity to the main road (Kuckuk et al. 2021)	Kuckuk et al. (2021)
Tenthredinidae	*Tomostethus nigritus*	1	*Fraxinus angustifolia, Fraxinus excelsior*	Higher temperatures, faster pest growth rate, soil compaction, disturbed hydrological conditions, large quantities of incorporated organic material, high salt concentration, Synchrony in the phenology of the insect with that of its host plant, Absence of flooding in cities, Increased density of larvae in urban environments, Pre-existing mechanical tree damage (Fons and Geert 2019)	Fons and Geert (2019)
Homotomidae	*Macrohomotoma gladiata* Kuwayama	1	*Ficus microcarpa*	None identified	Afechtal et al. (2021)
Gracillariidae	*Cameraria ohridella*	1	*Aesculus hippocastanum, Aesculus × carnea*	Water stress, urbanisation, traffic pollution (Dziegielewska et al. 2017)	Dziegielewska et al. (2017)
Tingidae	*Corythucha ciliata*	1	*Platanus × acerifolia, Platanus orientalis*	None identified	Georgieva et al. (2023)
Ortheziidae	*Praelongorthezia praelonga*	1	*Bauhinia variegata*	Water stress (Lemes et al. 2019)	Lemes et al. (2019)

##### Bacteria

Bacteria were represented by four families and affected seven tree species as well as multiple species in one of the publications (Table 4). Xanthomonadaceae bacteria caused foliage/needle discolouration, defoliation, the presence of chlamydospore masses, canker, and internal wood necrosis (Harris et al. 2014; Hashemi and Mohammadi 2016). Acholeplasmataceae were expressed as brown basal leaves, brown foliage, collapsing crowns (Ortiz-García et al. 2024), small or yellowing leaves, defoliation, tufted appearance foliage, dead branches, ‘witch’s broom’ (Filgueira et al. 2018), crown deformation, deliquescent internodes, abnormal elongation of apical shoots, leave pigmentation, epicormic growth, virescence and phyllody (Franco-Lara et al. 2017). Rhizobiaceae family was found to be causing foliage discolouration and shoot dieback (Lopes et al. 2010), dying crown, and thickened trunk with cankers (Tkaczyk et al. 2024). Water stress, xylem-feeding insect vectors, infected parental trees, and lack of regulation in production and commercialisation were found to be contributing to the distribution of bacteria and the infection levels (Franco-Lara et al. 2017; Harris et al. 2014; Lopes et al. 2010) (Table 4).

##### Arthropods

*Agrilus planipennis* (emerald ash borer) from the Buprestidae family was identified as a primary biotic factor causing dieback in Fraxinus species (Table 5). Symptomatic trees had exit holes on trunks, bark damage and bark loss, reduced foliage density, epicormic branching, scaffold cracks, branch fracture, and dieback (Persad and Tobin 2015; Selikhovkin et al. 2022; Straw et al. 2013; Volkovitsh et al. 2021). The effect of the activity of two other species of borers, this time from the Curculionidae family, were described by Coleman et al. (2019). In the USA, China, and Vietnam, maples, willows, and sycamores were infested by either of the insects, and, in some cases, it resulted in tree mortality rates exceeding 20%.

Gloomy scale *Melanaspis tenebricosa* from the Diaspididae family was found on Acer trees and observed causing the abundance of the pest and, consequently, canopy thinning, dead branches, darkened discoloured bark, and tree dieback (Backe et al. 2019; Dale and Frank 2014). Scale insects from the Coccoidea family affected multiple tree species, and as many as 96 new host species for these pests were recorded (de López et al. 2022). Trees attacked by these insects often showed high infestation levels, the presence of sooty moulds, yellowing of leaves, dried branches, and general dieback (Table 5).

Other pests were described in one publication each and included Aphididae, Tenthredinidae, Homotomidae, Gracillariidae, Tingidae, and Ortheziidae families which were found on Picea, Fraxinus, Ficus, Aesculus, Platanus, and Bauhinia trees (Afechtal et al. 2021; Dziegielewska et al. 2017; Fons and Geert 2019; Georgieva et al. 2023; Kuckuk et al. 2021; Lemes et al. 2019). The dieback in these cases was associated with water stress, higher temperatures, traffic pollution and elevated nitrogen in tree needles due to car traffic in particular, proximity to the main road, soil compaction, disturbed hydrological conditions, large quantities of incorporated organic material, high salt concentration, synchrony in the phenology of the insect with that of its host plant and faster pest growth rate, increased density of larvae in urban environments, pre-existing mechanical tree damage, and urbanisation in general (Table 5).

#### Contributing Factors

Many authors discussed factors contributing to tree decline (Tables 2–5). These were not tested but instead considered as a potential underlying driver that may be affecting urban trees. Climate, weather and water were the leading factors, followed by urban management, urbanisation, and soil properties. Others included tree size, structural problems, nutrients, and human activities.

**Fig. 4:**
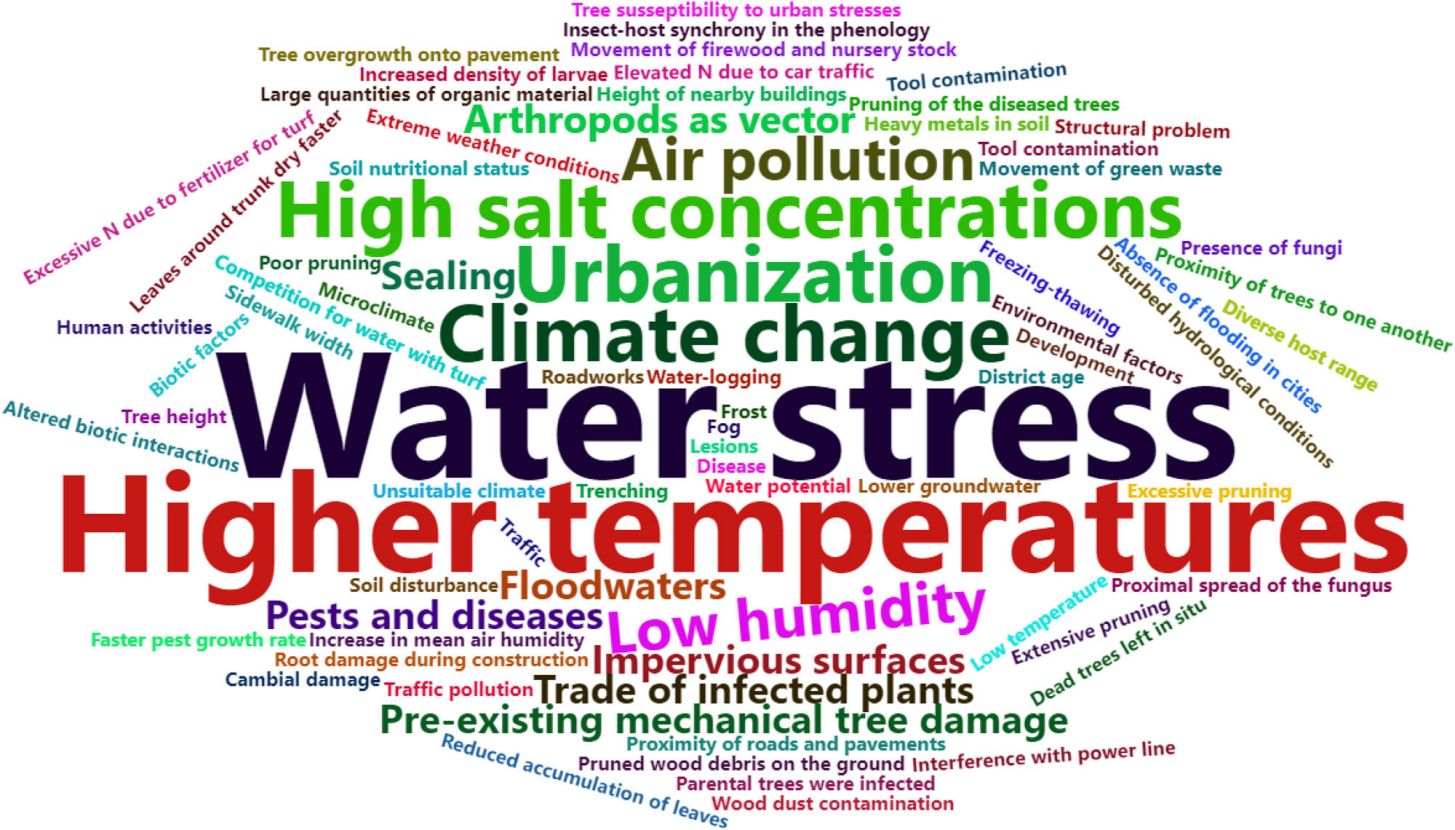
Words used to describe factors contributing to tree decline. Font size is proportional to the frequency of terms in the literature

Water limitation was identified as the number one problem that may lead to pathogen or pest outbreaks (Fig. 4). Aćimović et al. (2018) and Dziegielewska et al. (2017) referred to studies that point out that water stress is one of the main factors that result in the predisposition of trees to infection by opportunistic pathogenic fungi. Similarly, Backe et al. (2019) hypothesised that dry conditions may be contributing to the flare in gloomy scale populations. Water stress can also exacerbate the severity of a disease (Harris et al. 2014). Addressing the issue, in some cases, may result in a reduction or prevention of diebacks (Kovac et al. 2021).

Climate and weather, particularly unusual temperature extremes - either higher or lower than normal - appeared to be a common reason thought to contribute to tree decline. Betzen et al. (2021) and Marchin et al. (2022) suggested in their studies that heat stress results in sparse foliage, full leaf scorch or browning of leaf tips, crown dieback and necrosis in trees, as well as complete tree death. Miyamoto et al. (2019) also concluded that higher temperatures possibly played a role in the abundance of the bark beetle, which, in turn, caused extensive tree damage.

Soil compaction has been identified as a potential contributing factor in several studies, leading to the decline of trees and increased susceptibility to pathogens. Barber et al. (2013) emphasised that the development of a disease requires not only the presence of a pathogen and a host but also unfavourable environmental conditions, such as compacted soil. According to Dale and Frank (2014) and Miyamoto et al. (2019), soil compaction has the potential to weaken trees and possibly elevate water stress. Fons and Geert (2019) further discovered a strong correlation between soil compaction and the poor health of affected trees, suggesting that it may contribute to the outbreak of the ash sawfly (*Tomostethus nigritus*), along with other factors.

Low permeability in cities is another factor linked to tree decline. Dale and Frank (2014) and Jim (2005) suggested that impervious surfaces are problematic for street trees. Backe et al. (2019) found that the population of gloomy scale increased in trees, while Barber et al. (2013) noted that the number of beneficial mycorrhizal fungi decrease in such environments. At the same time, Volke et al. (2019) could not prove that impermeability has an effect on the infection rate by *Hymenoscyphus fraxineus*.

##### Urban Tree Management

**Fig. 5:**
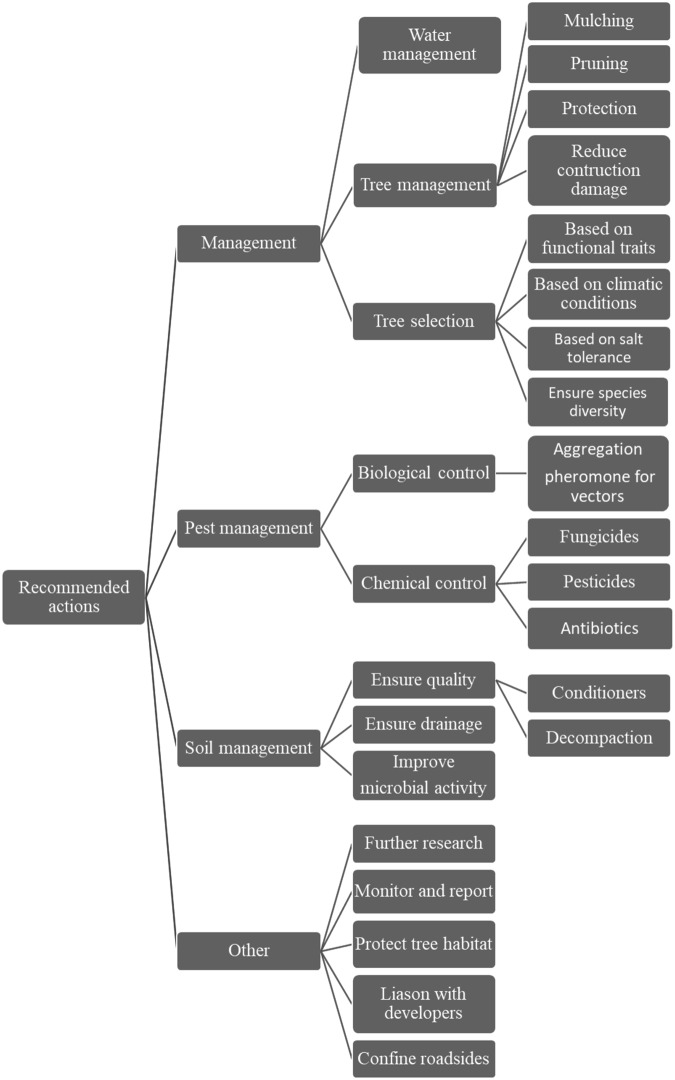
Management strategies and recommended actions proposed in the publications aiming to improve tree health and resilience in cities

Several recommendations on urban tree management were made by some of the authors (Fig. 5). Several of them emphasised the importance of adequate water management (Acimovic et al. 2018; Backe et al. 2019; Kovac et al. 2021; May et al. 2013; Pelleteret et al. 2017; Persad and Tobin 2015; Yeganeh and Mohammadi 2022), tool sanitation (Fiorenza et al. 2022; Yeganeh and Mohammadi 2022), mulching (Acimovic et al. 2018; Barber et al. 2013; Kovac et al. 2021), and pruning (Acimovic et al. 2018; Fiorenza et al. 2022; Kovac et al. 2021). Changes in the tree selection parameters also received some attention, with recommendations for selection based on functional traits, suitability for climate, and resilience to environmental stresses (Marchin et al. 2022; Snieskiene et al. 2016; Volke et al. 2019). It was also suggested that it is important to ensure species diversity, species replacement in case of failure, and timely tree removal (de López et al. 2022; Franco-Lara et al. 2017; Kuckuk et al. 2021).

Soil management was also considered in some studies, though to a lesser extent. Only five papers out of 65 addressed the challenges faced by soil as a habitat for trees. The authors emphasised the importance of soil quality, including proper drainage and permeability, which can be enhanced through decompaction and the use of soil conditioners and amendments (Acimovic et al. 2018; Barber et al. 2013; Jim 2005).

Since many authors found that pathogens or pests were responsible for tree decline, the use of biological and chemical control was suggested. These included treatments with antibiotics, fungicides, or pesticides. The use of pheromone to trap vector beetles was proposed in one study, and insect management was recommended to be done at the time of tree pruning (de López et al. 2022; Filgueira et al. 2018; Fiorenza et al. 2022; Kovac et al. 2021; Kuckuk et al. 2021; Miyamoto et al. 2019; Ortiz-García et al. 2024; Yeganeh and Mohammadi 2022).

## Discussion

This review examined a range of factors influencing urban tree health, including biotic and abiotic elements, as well as their interactions. It was found that some tree species are affected more than others, and while the native species were represented more than the introduced ones, there was no indication from the authors that it may be affecting their survival. Trees in the Mediterranean, humid subtropical, and humid continental climates were found to be affected more, possibly due to the higher temperatures and humidity. Although tree pests and pathogens were often identified as the primary causes of decline, other factors frequently contributed to increased susceptibility to disease. As highlighted by Morkunas et al. (2018), exposure to an initial stressor can impair a tree’s defence mechanisms, thereby reducing its ability to respond effectively to subsequent stressors.

*Platanus, Fraxinus, Acer*, and *Ficus* - widely distributed and economically important trees - were the top genera reported declining. While being quite hardy and tolerant to harsh conditions, they are affected by urbanisation, site and climatic conditions, as well as pests and pathogens (Enderle et al. 2019; Gregorová et al. 2010; Kamer Aksoy 2022). Careful modelling for these, as well as other commonly declining trees, is required to predict further diebacks and to develop clear management strategies in urban areas.

According to the 2023 IPCC report, it is expected that the current global warming will further alter the global water cycle with more frequent heatwaves and droughts (IPCC, 2023), which will be more intense in urban areas. Additionally, constant human migration and the movement of goods increase the likelihood of pathogen spread between cities, thereby heightening the vulnerability of urban trees to disease (Santini et al. 2018). This suggests a greater risk of tree dieback due to the combined effects of abiotic and biotic factors that will be experienced in the future.

### Abiotic Threats and Their Management

Abiotic threats received less attention in the presented studies compared with pathogens and pests, yet they were often discussed as possible contributors or underlying factors. The most frequently mentioned problems, main as well as predisposing, were water availability, temperature, climate, urbanisation, soil compaction, and high soil salinity.

Water limitation and heat stress can be addressed in cities, to a degree. In countries with irregular or low rainfall or areas where climatic changes shift the weather to more dry patterns, irrigation might be necessary for vulnerable trees. This is crucial, as drought stress depresses photosynthesis (Wang et al. 2019), reduces growth (Franceschi et al. 2023), increases the risk of cavitation, possibly due to hydraulic deterioration (Savi et al. 2015), and increases vulnerability to pathogen and pest attacks (Dale and Frank 2017; Tubby and Webber 2010). Various water intake improvement designs are available for use, from passive (Thom et al. 2022) to smart irrigation systems, allowing to lower water usage while supporting trees (Gimpel et al. 2021).

On the other hand, increasing chances of floods (IPCC, 2023) and, therefore, untypical prolonged inundation of trees close to the water bodies are expected, which many species may not survive. While increasing the number of green infrastructure elements such as green roofs, swales, and rain gardens is important for slowing down and reducing the run-off, they do not fully fix the problem and may not be enough (Qin 2020). Therefore, to increase chances of survival in areas with a potential risk of flooding, trees from different ecological habitats that can withstand at least one or more environmental stresses should be selected (Leksungnoen 2017).

Higher temperatures are more difficult to address and, as a minimum, require clear communication and collaboration between the tree owners and urban planners to reduce the effects of UHI. This can be done by lowering surface temperatures through improving plant spatial configuration (Rakoto et al. 2021), increasing proximity to pavement (Chen et al. 2017), selecting pavement colour and texture with minimal contribution to UHI (Senevirathne et al. 2021), and taking into consideration the height of the buildings and urban density to control airflow and temperatures (Xi et al. 2021; Xue et al. 2020).

Soil condition was identified as one of the factors influencing tree health, with compaction being the biggest contributor. Compaction negatively affects tree volume (Norris et al. 2014), reduces the air-filled porosity and infiltration of the soil, and simultaneously increases water runoff, leading to dehydration in trees (Yang and Zhang 2011). It also increases with the proximity of footpaths due to trampling (Kostrakiewicz-Gieralt et al. 2022) and with frequent grass mowing (Foshee et al. 1997), when heavy machinery is used. To add complexity, Wilkaniec et al. (2018) found that trees are more prone to insect and pathogen attacks if they are located in proximity to a heavily trafficked route.

In countries with colder climates, sodium chloride is often used for de-icing. Marosz (2011) found that closer to de-iced roads, soil has higher electrical conductivity, and trees accumulate high levels of sodium ions in the leaves, which impacts their growth. Cekstere et al. (2008) found a correlation between the concentration of salts and leaf necrosis along the roads. High salinity not only alters soil’s physical and chemical properties, but it is also known to negatively affect the structure, function, and diversity of microbial communities (Singh 2016). Hence, finding good alternatives to sodium chloride might be necessary for tree protection.

Careful tree selection with weather extremes, chances of hurricanes, and potential flood events in mind could assist in tree survival. Those advocating for greater attention to species selection acknowledge the complexity of this task and its location-specific nature (Vogt et al. 2017). When planning street and park tree planting, it is imperative to ensure that the chosen species not only align with the climatic zone but also possesses traits conducive to survival in urban environments (Yang 2009).

### Biotic Threats

While there was a great variation in pathogens and pests discovered in the studies, a few deserve close attention due to the frequency in appearance or severity of the effects.

The most commonly found pathogens in trees were from the Botryosphaeriaceae family, a highly diverse fungus (Botryosphaeriales, Ascomycetes). This species can act as saprobic, endophytic, or a latent pathogen (Phillips et al. 2013; Slippers and Wingfield 2007) and can be aggressive and kill its host, especially if it is already predisposed by unfavourable environmental conditions. Botryosphaeriaceae travel via spores and can infect seeds, foliage, bark, or fruit of a tree via natural openings and their activity is linked to drought, physical damage, other pathogens, and unsuitable site selection (Slippers and Wingfield 2007). Many Botryosphaeriaceae fungi do not have a specific host (Phillips et al. 2013), which makes it easier for them to spread. Trees do not have to be wounded or damaged for the fungus to infect due to it being endophytic (Slippers and Wingfield 2007), yet wounding and stress are known to trigger the spread of infection (Darge and Woldemariam 2021; van Niekerk et al. 2011).

Trees can be protected from the biotic threat by ensuring water availability, the improvement of the ventilation of the canopy, pruning in dry weather, and tree surgery (Moral et al. 2019; Serrano et al. 2015). Alternatively, various tree fungicide controls are available to treat Botryosphaeriaceae species (Billones-Baaijens et al. 2015; Brown-Rytlewski and McManus 2000), although the use of chemicals should be considered only as a last resort, as their application can disrupt ecosystems by impacting non-target species. Research has shown that fungicide use can, for example, harm fungal and mite communities (Schaeffer, 2017; Shinichi and Katayama 2021), impair lichen physiology (Rola et al. 2023), and stunt the growth of some nematodes (Bradford et al. 2020). Addressing symptoms may provide a quick fix but thinking long-term means equipping trees with defence mechanisms so they can protect themselves.

Another pathogen that deserves the attention of urban tree managers is mould species from the genus *Phytophthora*, notorious soil-born pathogens that cause root rot (Jung et al. 2017; Wickland et al. 2008). There have not been many records of it in urban areas in the literature, but these species are widespread and generally known to kill trees relatively quickly (Barber et al. 2013) and may cause problems in the future. Some fungicides are found to be effective in the treatment of Phytophthora and inhibit its growth, although the pathogen can become tolerant and less susceptible to the treatment (Hunter et al. 2022). Quarantine is another way to stop the spread of the disease and is possibly the most effective approach (Hansen 2015).

As a number of papers looked at the dieback of Fraxinus (ash tree), *Agrilus planipennis* (emerald ash borer) was found to be a common pest of urban trees. This invasive species is native to East Asia, possibly ‘hitchhiking’ via vehicles and is responsible for the mass decline of ash in North America, Russia, and some parts of Europe (Selikhovkin et al. 2022; Valenta et al. 2017). *A. planipennis* is found to be more attracted to stressed trees (Crook and Mastro 2010), therefore, once the insect has made its way in, the risk of infestation in cities can be quite high. Different types of insecticides have been used to treat the borer (McKenzie et al. 2010), and the success largely depends on whether early intervention was possible, the proportion of trees that were treated, and whether those trees were closely located (McCullough and Mercader 2012). While all ash trees can be colonised by the beetle, some species are more resistant, and others are highly vulnerable to the invasion (Tanis and McCullough 2015).

On top of the already mentioned problems, urbanisation often alters soil structure, leading to changes in its quality, including modifications in structure, organic matter levels, and nutritional values (Santorufo et al. 2021). Abundant evidence supports the significance of maintaining not only soil moisture but also adequate nutrient levels as a preventative strategy for enhancing pathogen resistance. In a comprehensive review, Dordas (2008) highlighted the importance of macro- and micronutrients, such as nitrogen, potassium, phosphorus, manganese, zinc, boron, chlorine, and silicon, in controlling plant diseases. While nutrients alone cannot eliminate pathogens, they play a crucial role in reducing disease levels, providing a foundation for further treatment with appropriate biocides.

The effect of soil salinity on pathogens can be ambiguous. It may impact plant disease distribution and severity, either in a positive or negative way. These may include the reduction in sporulation in fungi, yet an increase in pathogenic virulence. A combination of high salinity and pathogenic activity can be detrimental to plant health (Dikilitas and Karakas 2014).

Many papers advocate for greater attention to species selection, acknowledging the complexity of this task and its location-specific nature (Vogt et al. 2017). When selecting street and park trees, it is imperative to adopt a proactive adaptation approach, ensuring that chosen species not only align with the climatic zone but also possess traits conducive to survival in urban environments (Yang 2009).

Tree and soil management were frequently discussed in the selected papers. While no direct connections were made to the decline in the majority of the studies, a clear message was that the correct management of urban forests can support the longevity and health of trees. Monitoring, appropriate tree selection, and supplemental watering received the most attention in the literature.

‘Monitoring’ emerged as the most frequently referenced aspect, highlighting the significance of maintaining control over tree health in urban areas. While the use of satellite or drone imagery for tree assessment is not novel in forestry and crop production, its adoption in urban settings is also gradually increasing. This approach proves to be suitable for large urbanised areas, offering cost-effectiveness and simplicity while yielding results comparable to on-the-ground assessments (Degerickx et al. 2018; Klobucar et al. 2021). Diversification can help avoid catastrophic losses to pathogens and pests (Raupp et al., 2006), while mistakes in the selection process can lead to ecological and economic costs (Vogt et al. 2017).

### Knowledge Gaps

This review synthesised research regarding the threats to urban trees. Only peer-reviewed case studies were selected as part of this study, of which there were a limited amount in the scientific literature. It is acknowledged that information about tree dieback is often not recorded or recorded in governmental reports of variable quality or accessibility and that future studies might include social research methods to capture some of this qualitative data from urban tree management practitioners to supplement the scientific literature. This literature review could help form the basis of surveys and interviews for attaining these critical insights.

Tree decline is frequently unseen until it is non-reversible, often after prolonged periods of extreme weather events. In cities with well-developed urban forests, monitoring can be expensive, and adoption of the analysis of satellite and drone imagery may be necessary, combined with on-the-ground observations. Finally, a clear understanding of how soil- and airborne pathogens threaten each climatic zone and species and how their distribution changes with climate change and globalisation can be crucial for the development of management strategies and dieback prevention.

### Study Limitations

Two search engines, Web of Science and Scopus, were used for this review. Neither produced outputs relevant to the topic for the years prior to 2000. Although the authors of this manuscript acknowledge that significant research was conducted in the 20th century - such as studies of problems caused by engineering (Foster and Blaine 1978), compaction (Jim 1993), issues such as natural gas leakages in soil, air pollutants, and heavy metals (Impens and Delcarte 1979), or pests (Ball and Simmons 1980), there was no literature that met the search criteria prior to 2001.

## Conclusion

Urban trees face numerous challenges, including factors like water limitations, poor soil quality and compaction over which we have some control, as well as uncontrollable elements such as extreme weather events. There is an increasing interest in comprehending these factors to foster resilient and thriving urban forests as cities grapple with the effects of climate change, increasing densification, and the loss of green space. However, further research is essential to inform effective management strategies. This review of 65 articles highlights the necessity for (I) integration of preventative measures such as soil care into urban tree management rather than post-treatment approaches, (II) better understanding of local pests and pathogens and their hosts, (III) implementation of a systematic approach to tree selection in cities based on the knowledge of traits and threats, and (IV) building of urban forests with climate change and potential extreme weather events in mind. All of these require careful planning and modelling, with the goal of creating a comfortable and healthy environment for the current and next generations of those living in cities.

## Data Availability

No datasets were generated or analysed during the current study.
